# Vitamin D and Calcium Supplementation Accelerate Vascular Calcification in a Model of Pseudoxanthoma Elasticum

**DOI:** 10.3390/ijms23042302

**Published:** 2022-02-19

**Authors:** Elise Bouderlique, Ellie Tang, Jeremy Zaworski, Amélie Coudert, Dominique Bazin, Ferenc Borondics, Jean-Philippe Haymann, Georges Leftheriotis, Ludovic Martin, Michel Daudon, Emmanuel Letavernier

**Affiliations:** 1 UMR S 1155, Sorbonne Université, 75020 Paris, France; elise.bouderlique@inserm.fr (E.B.); ellie.tang@inserm.fr (E.T.); jeremy.zaworski@inserm.fr (J.Z.); jean-philippe.haymann@aphp.fr (J.-P.H.); micheldaudon24@gmail.com (M.D.); 2 Institut National de la Santé et de la Recherche Médicale (INSERM), Sorbonne Université, 75020 Paris, France; 3 UFR d’odontologie (Département des Sciences Biologiques), Université Paris Diderot BIOSCAR—INSERM U1132, Hôpital Lariboisière, 75010 Paris, France; amelie.coudert@inserm.fr; 4 Laboratoire de Physique des Solides, CNRS UMR 8502, Université Paris Sud XI, 91405 Orsay, France; dominique.bazin@u-psud.fr; 5 Synchrotron Soleil, L’Orme des Merisiers, Saint-Aubin–BP48, CEDEX, 91192 Gif-sur-Yvette, France; ferenc.borondics@synchrotron-soleil.fr; 6 Physiology Unit, AP-HP, Hôpital Tenon, 75020 Paris, France; 7 Laboratory of Physiology and Molecular Medicine (LP2M), CNRS-UNS UMR 7370, University of Nice, 28 rue de Valombrose, CEDEX 2, 06107 Nice, France; leftheriotis.g@chu-nice.fr; 8 MITOVASC Institute—UMR CNRS 6015 INSERM U1083 Angers University, 49100 Angers, France; lumartin@chu-angers.fr; 9 PXE Consultation Center, MAGEC Reference Center, MAGEC Nord Center for Rare Skin Diseases, Angers University Hospital, 49100 Angers, France

**Keywords:** vitamin D, calcium, calcification, vascular, pseudoxanthoma elasticum, pyrophosphate, ABCC6

## Abstract

Arterial calcification is a common feature of pseudoxanthoma elasticum (PXE), a disease characterized by *ABCC6* mutations, inducing a deficiency in pyrophosphate, a key inhibitor of calcium phosphate crystallization in arteries. Methods: we analyzed whether long-term exposure of Abcc6^−/−^ mice (a murine model of PXE) to a mild vitamin D supplementation, with or without calcium, would impact the development of vascular calcification. Eight groups of mice (including Abcc6^−/−^ and wild-type) received vitamin D supplementation every 2 weeks, a calcium-enriched diet alone (calcium in drinking water), both vitamin D supplementation and calcium-enriched diet, or a standard diet (controls) for 6 months. Aorta and kidney artery calcification was assessed by 3D-micro-computed tomography, Optical PhotoThermal IR (OPTIR) spectroscopy, scanning electron microscopy coupled with energy-dispersive X-ray spectroscopy (SEM-EDS) and Yasue staining. Results: at 6 months, although vitamin D and/or calcium did not significantly increase serum calcium levels, vitamin D and calcium supplementation significantly worsened aorta and renal artery calcification in Abcc6^−/−^ mice. Conclusions: vitamin D and/or calcium supplementation accelerate vascular calcification in a murine model of PXE. These results sound a warning regarding the use of these supplementations in PXE patients and, to a larger extent, patients with low systemic pyrophosphate levels.

## 1. Introduction

Arterial calcification is a feature of aging, atherosclerosis, chronic kidney disease, and diabetes [1]. A few monogenic disorders are characterized by an early-onset of vascular calcification affecting the arterial media [2,3,4]. These diseases include pseudoxanthoma elasticum (PXE), the most frequent one, generalized arterial calcification of infancy (GACI), and arterial calcification due to deficiency of CD73 (ACDC). These three conditions promote a deficiency in pyrophosphate, a key inhibitor of calcium phosphate crystallization in soft tissues and arteries [2,3,4].

PXE results from biallelic mutations of the *ABCC6* gene, encoding for ABCC6 (ATP-binding cassette, subfamily C member 6), a transmembrane ABC transport protein expressed mainly in the liver and the kidney. PXE patients are affected by vascular (arterial), retinal, kidney, and skin calcifications [2,3,5,6,7]. ABCC6 promotes adenosine triphosphate (ATP) externalization in the blood, where ATP is hydrolyzed by the ectonucleotide pyrophosphatase phosphodiesterase-1 (ENPP1), inducing systemic pyrophosphate and adenosine monophosphate (AMP) release. ABCC6 and ENPP1 are actually the main source of systemic pyrophosphate, limiting ectopic hydroxyapatite crystalline deposits in arteries and other tissues. Patients with PXE, as well as Abcc6^−/−^ mice, have a reduced plasma pyrophosphate level which explains at least partly their mineralization disorder [2,3,4,6,7].

The deficiency in pyrophosphate increases calcium phosphate supersaturation and arterial calcification initially localized at the elastic fibers of the medial layer, in the medium- and small-sized musculo-elastic arteries. The arterial calcification affecting PXE patients is similar to the calcification observed in chronic kidney disease and diabetes and, interestingly, patients with advanced CKD also have low pyrophosphate serum levels [8,9]. Patients affected by ENPP1 mutations develop GACI, causative for very low systemic pyrophosphate levels and life-threatening vascular calcification. Deficiency of CD73 (ACDC) increases TNAP activity, which decreases pyrophosphate systemic concentration, and therefore also results in dramatic systemic vascular calcification [2,3,4].

The role of vitamin D and calcium intakes in vascular calcification is controversial. On the one hand, vitamin D may increase calcium phosphate supersaturation in blood by promoting intestinal calcium absorption and decreasing renal phosphate excretion through repression of parathyroid hormone synthesis [10]. Experimental animal models evidenced that vitamin D dramatically accelerates arterial calcifications but the doses of vitamin D were often very high in these experiments (100,000 to 500,000 IU/Kg/day) [11,12,13]. On the other hand, vitamin D might exert anti-inflammatory effects, which are potentially beneficial in vascular diseases [10,14].

We previously demonstrated that vitamin D, especially when combined with increased calcium intakes, can accelerate the development of renal papillary calcification (the first step toward kidney stone formation) in Abcc6^−/−^ mice in a long-term model [15]. We took the opportunity presented by this model to analyze the impact of vitamin D and calcium on large and small arteries. Actually, vitamin D is widely prescribed, and because PXE patients are affected by an increased calcium phosphate supersaturation in blood (due to the low pyrophosphate level), our aim was to identify whether moderate doses of vitamin D and/or calcium intakes would accelerate arterial calcification in a long-term murine model of PXE (Abcc6^−/−^ mice).

## 2. Results

### 2.1. Mice Survival, Weight and Biological Results

Weight was slightly higher in wild-type animals than in Abcc6^−/−^ mice at the end of the protocol but remained similar among the four Abcc6^−/−^ groups. There was no weight loss in any group during the protocol (not shown). One wild-type mouse exposed to calcium and vitamin D died for an unknown reason.

As previously published, there was no significant difference between serum calcium and phosphate levels observed between the different groups at 6 months [15]. As expected, Vitamin D serum levels increased mildly (×1.5 control serum levels) in the groups receiving vitamin D (ergocalciferol) supplementation (for instance, 124.4 ± 5.8 ng/mL in Abcc6^−/−^ mice exposed to vitamin D vs. 88.9 ± 6.9 ng/mL in control Abcc6^−/−^ mice, *p* < 0.05). Serum pyrophosphate levels were difficult to assess in blood but urinary pyrophosphate excretion was, as expected, decreased in Abcc6^−/−^mice (Abcc6^−/−^ vs. wild-type mice at each time point; *p* < 0.01). [15]. Of note, urinary pyrophosphate excretion was not significantly modified by calcium and/or vitamin D among Abcc6^−/−^ mice [15].

### 2.2. Aorta Calcifications

**Figure 1:**
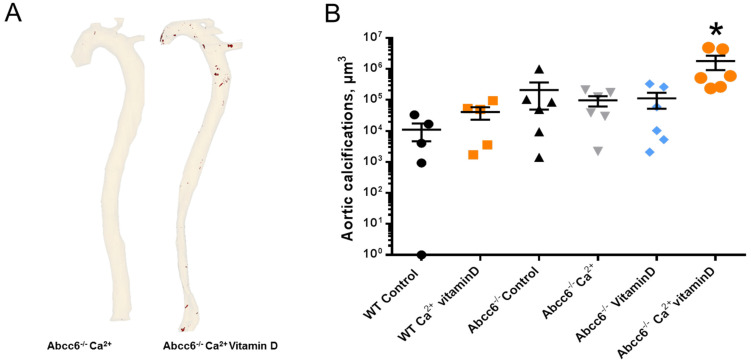
(**A**). Representative 3D reconstructions of micro-CT analyses in aorta of two Abcc6^−/−^ mice exposed to, respectively, high-calcium diet and high-calcium + vitamin D for 6 months. Calcifications (high-density areas) appear in red. (**B**). Volume of aorta calcification quantified by CT-scan in WT and Abcc6^−/−^ mice from all groups. * *p* < 0.05 vs. all other groups, Mann-Whitney test.

**Figure 2:**
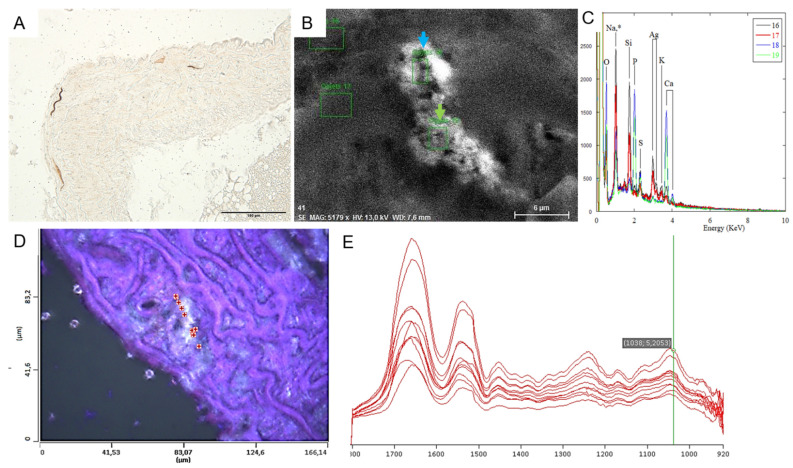
(**A**). Yasue staining of an aorta (Abcc6^−/−^ mouse exposed to high-calcium + vitamin D) showing that incipient calcification, stai ned in black, seem localized in elastic fibers. (**B**,**C**). Scanning electron microscopy coupled with EDS evidencing the presence of high amounts of calcium and phosphate in specific areas, at the surface of elastic fibers, corresponding to arrows (**B**) and blue and green peaks (**C**). EDX spectra corresponding to area 16,17,18, and 19 including the different contributions coming from the support and the sample namely O (K_α_ = 0.524 KeV), Na (K_α_ = 1.041 KeV), Si (K_α_ = 1.740 KeV), P (K_α_ = 2.013 KeV), S (K_α_ = 2.307 KeV), Ag (L_α_=2.985 KeV, L_β_ = 3.150 KeV), K (K_α_=3.310 KeV) and Ca (K_α_=3.690 KeV, K_β_ = 4.010 KeV). Spectra collected for the support show that Ca and P belong to the calcifications while other elements belong to the support (**D**,**E**). mIRage technique characterizing incipient calcifications localized in the close vicinity of elastic fibers. The spectra reveal the presence of different absorption bands of the apatite including the ν_3_ P-O stretching vibration mode measured at 1035–1045 cm^−1^. Carbonate ions are detected together with apatite by their ν_3_ C-O stretching vibration mode around 1420 cm^−1^. Each dot in the upper panel corresponds to one spectrum.

- Micro-CT analyses and 3D reconstructions were performed to assess the topography and measure the global volume of aorta calcifications (Figure 1). Wild-type mice did not develop significant calcifications, even after exposure to calcium and vitamin D. Abcc6^−/−^ mice spontaneously developed very mild calcifications at the time of the sacrifice (8 months-old animals) but only Abcc6^−/−^ mice exposed to both calcium and vitamin D had a significant increase in aortic calcification volume, in comparison to unexposed Abcc6^−/−^ mice (Figure 1, * *p* < 0.05 vs. all other groups, Mann-Whitney test).

- Histopathological analyses and Yasue staining evidenced that calcifications were initiated in the close vicinity of elastic fibers in the aortic media, as observed in PXE patients, and were not atherosclerotic lesions (Figure 2). To go further in the characterization of the crystalline phases, we gathered observations through scanning electron microscopy coupled with energy-dispersive X-ray spectroscopy (SEM-EDS), evidencing that deposits on elastic fibers contained high amounts of calcium and phosphate (Figure 2). OPTIR experiments using the mIRage experimental setup identified that incipient calcifications were made of carbonated apatite (Figure 2). The analysis of the spectra revealed some features specific to the presence of different absorption bands of biological apatite which has the general chemical formula Ca_10−x_□_x_(PO_4_) _6−x_(CO_3_)_x_(OH)_2−x_□_x_ with 0 < x < 2), including the ν_3_ P-O stretching vibration mode measured at 1035–1045 cm^−1^ (Figure 2). Carbonate ions were detected together with apatite by their ν_3_ C-O stretching vibration mode around 1420 cm^−1^.

### 2.3. Kidney Arteries Calcifications

**Figure 3:**
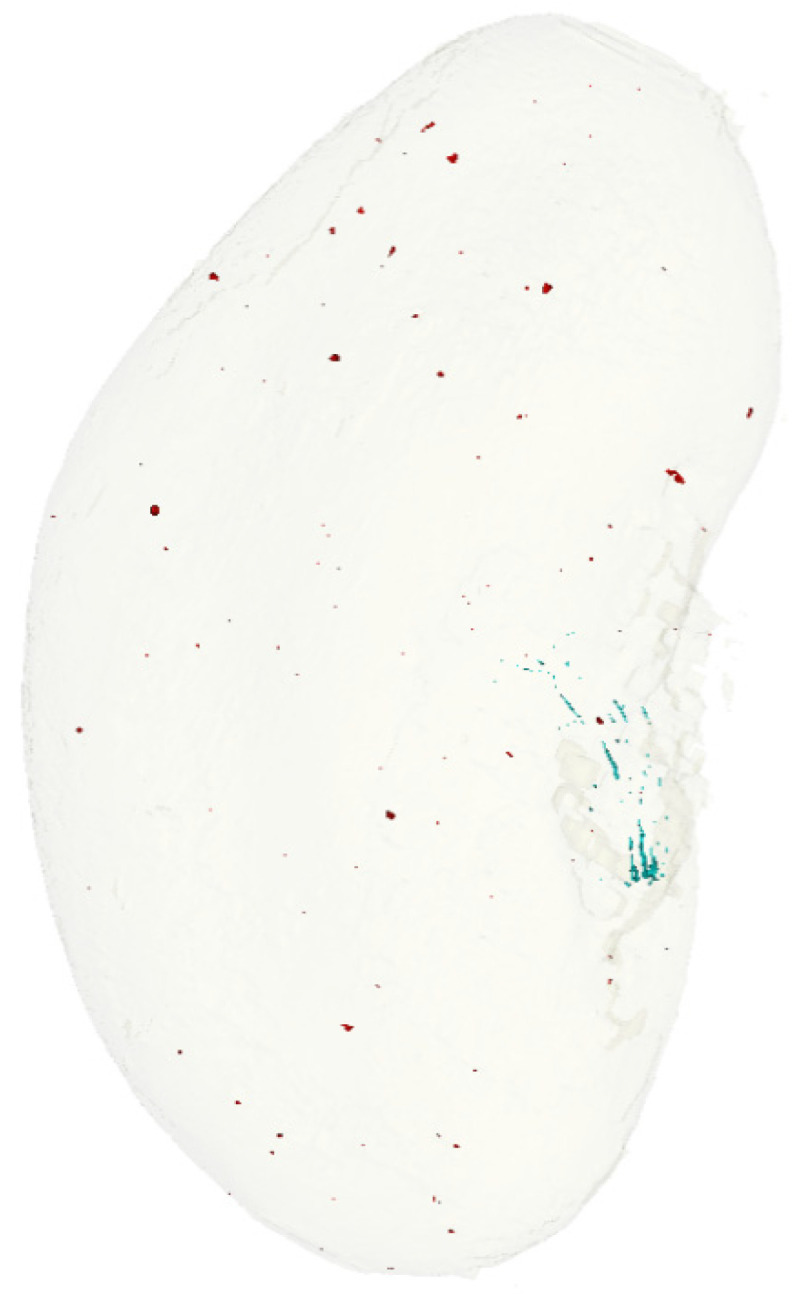
Representative 3D reconstruction (micro-CT) of one kidney from an Abcc6^−/−^ mouse exposed to high-calcium + vitamin D for 6 months. Calcifications (high-density areas) were stained in blue in the papilla (Randall’s plaque) and in red in the cortical and medullary areas.

**Figure 4:**
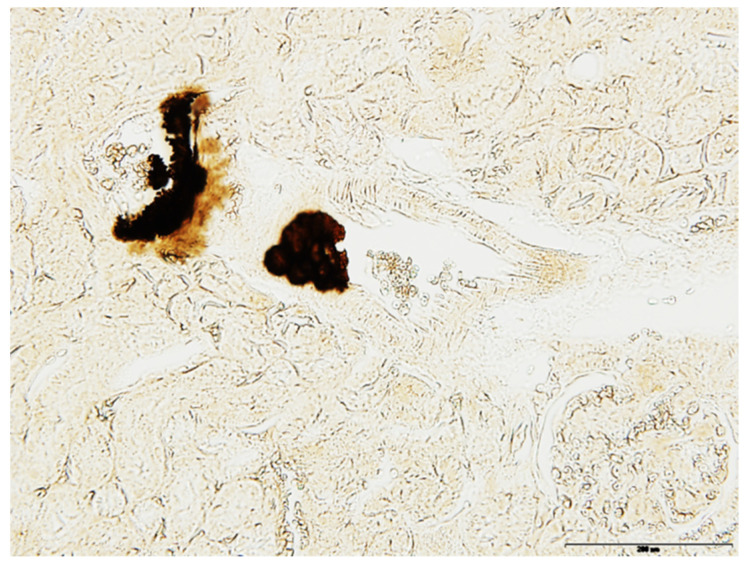
Representative kidney calcifications stained with Yasue method, evidencing arterial calcifications (in the vascular wall).

**Figure 5:**
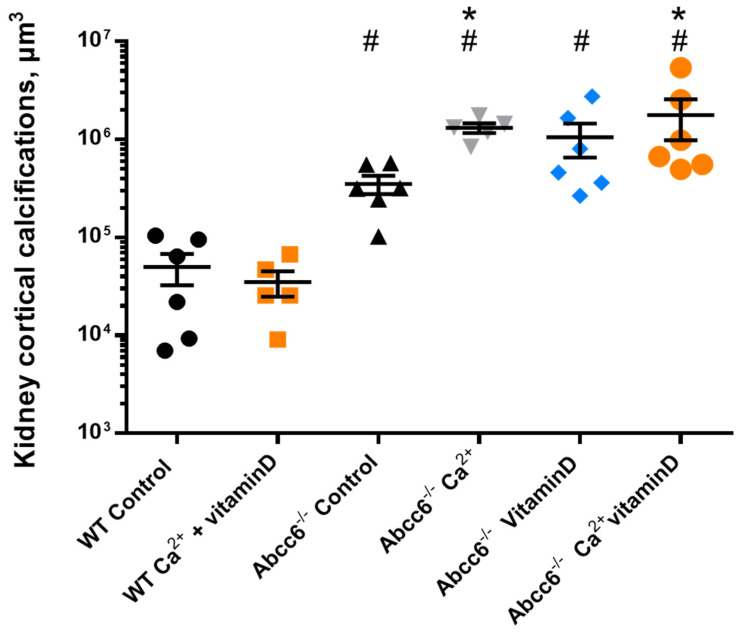
Volume of kidney vascular calcification quantified by CT-scan in WT and Abcc6^−/−^ mice from all groups. * *p* < 0.05 vs. Abcc6^−/−^ control (untreated) mice. # *p* < 0.05 vs. WT control or WT Ca2^++^ vitamin D mice.

- Micro-CT analyses and 3D reconstructions were performed to assess the topography and measure the global volume of small-sized/medium-sized renal artery calcification (Figure 3). CT-scans of kidneys revealed 2 sites of calcifications: Randall’s plaque at the tip of the papilla, previously described, and spotty calcifications in the cortical area (Figure 3) [15].

- Histological examination and Yasue staining of kidneys revealed that cortical calcifications were actually arterial calcifications (Figure 4). The use of µFTIR techniques also evidenced that these calcifications were made of carbonated apatite, with typical absorption bands.

- Cortical calcifications were measured independently from papillary calcification (exclusion of the papillary area), to assess only vascular calcification volume. Wild-type mice did not develop significant kidney arterial calcifications, even after exposure to calcium and vitamin D (Figure 5). Abcc6^−/−^ mice developed spontaneously very mild calcifications at the time of the sacrifice (8 month-old animals) but CT-scan quantification of these cortical calcifications showed that calcium and calcium + vitamin D enriched diets significantly increased kidney arterial calcifications in Abcc6^−/−^ mice (* *p* < 0.05 vs. Abcc6^−/−^ control mice, Mann-Whitney test). (Figure 5).

## 3. Discussion

The long-term administration of vitamin D, in synergy with calcium supplementation, dramatically accelerated the development of aorta calcification in Abcc6^−/−^ mice. When focusing on small arteries in a specific organ (kidney), calcium with or without vitamin D supplementation accelerated the development of vascular calcification in these mice. Of note, exposure of wild-type mice to calcium and/or vitamin D supplementation did not promote the development of calcification [15]. As previously published, mice exposed to calcium and/or vitamin D had no significant changes in calcium and phosphate serum levels.

Vascular calcification is characterized by the development of calcium phosphate (carbonated apatite) crystal deposition in vascular walls. Various forms of calcification have been described: intimal calcification is associated with atherosclerotic lesions and may be distinguished from medial calcification, even if both types may be associated in an individual [1,16]. Medial calcification is an important feature of the vascular disease observed in chronic kidney disease (CKD) and in diseases characterized by systemic pyrophosphate deficiency (PXE but also GACI and ACDC) [2,3,4,6]. Patients affected by these conditions have an increased risk of cardiovascular events and mortality. In these populations, the “classical” cardiovascular risk factors (hypertension, tobacco, dyslipidemia, diabetes, etc.) do not explain the development of vascular calcification, suggesting a role for non-traditional risk factors, including disordered mineral metabolism [2].

Calcium phosphate crystallization in tissues is promoted by nucleation processes (local factors decreasing the energy necessary for crystallization) and by increased calcium phosphate supersaturation, at a local or systemic level. Calcium phosphate supersaturation results from an increase in promoters of calcification, i.e., increased phosphate and/or calcium concentration or from a decrease in calcification inhibitors, i.e., pyrophosphate, fetuin A, Matrix Gla Protein, osteopontin, and many others [16,17]. The tissue non-alkaline phosphatase (TNAP) is a major determinant of physiological and pathological calcification and illustrates this aspect: the enzyme degrades pyrophosphate in inorganic phosphate, increasing dramatically local supersaturation in calcium phosphate by decreasing the inhibitor and increasing the concentration of the promoter. It has been shown that pyrophosphate deficiency resulting from alkaline phosphatase activity during vascular remodeling increases biomineralization [9]. In patients affected by CKD, high serum phosphate levels increase the calcium-phosphate supersaturation and are associated with vascular calcification and cardiovascular mortality. Interestingly, a deficiency in pyrophosphate has also been described in CKD patients and may play an important role in the development of cardiovascular calcification [8,18]. Calcium phosphate supersaturation is probably necessary for the development of vascular calcification but phosphate, and to a lesser extent calcium, may also exert an active function on vascular smooth muscle cells by changing their phenotype into osteoblast-like cells expressing RUNX2 and other osteogenic genes, increasing mineralization in arterial media [19].

The use of CT-scans allowed quantification of the amount of calcification in tissues but the resolution was not sufficient to identify the origin of the process. We observed in many arteries, including the aorta, small incipient calcifications located in the media. The use of Field emission-scanning electron microscopy coupled with energy dispersive X-ray analyses and of the mIRage technique provided a good resolution showing that apatite deposits were in close contact with elastin fibers. These results confirm that calcifications induced in our models affected arterial media and were not atherosclerotic lesions.

The predominant function of vitamin D is to regulate calcium and phosphate homeostasis, but it was also reported to exert pleiotropic effects on various organs including the cardiovascular system. Vitamin D increases calcium intestinal absorption, and may also affect phosphate homeostasis by increasing intestinal phosphate absorption and decreasing parathyroid hormone synthesis [20]. In animal models, administration of vitamin D is a classic method to promote the development of cardiovascular calcification but the doses used are usually extremely high and causative for hypercalcemia. For instance, the administration of 500,000 IU/kg/day vitamin D for three consecutive days in mice promotes severe medial calcifications after 7 days [11]. Administration of the active form of vitamin D, calcitriol, also results in massive vascular calcification in rats [12]. In these animal models, vitamin D-induced calcification was related to an increase in serum calcium and phosphate levels, increasing calcium-phosphate supersaturation dramatically. Moreover, a genomic effect of calcitriol through its receptor VDR has been proposed, its activation increasing mineralization by vascular smooth muscle cells in vitro [21]. On the other hand, vitamin D may exert pleiotropic effects that may be beneficial in cardiovascular diseases [10]. Actually, in experimental models, vitamin D activates VDR in vascular endothelial cells, regulates the renin-angiotensin-aldosterone system, and exerts anti-inflammatory effects [22].

In clinical settings, a correlation between vitamin D serum levels and cardiovascular events was described: low vitamin D levels (usually <20 ng/mL) were linked to an increased risk of myocardial infarction stroke, cardiovascular mortality, and heart failure in case-control studies [23]. Based upon these epidemiological observations, a large number of prospective randomized controlled trials were conducted during the past years to assess whether vitamin D supplementation would improve cardiovascular outcome. The results were negative; vitamin D supplementation was not associated with reduced major adverse cardiovascular events, myocardial infarction, stroke, cardiovascular mortality or all-cause mortality [23]. As stated in a recent meta-analysis, these findings suggest that vitamin D supplementation does not confer cardiovascular protection and is not indicated for this purpose [24]. These results suggest that, as observed in other diseases, low vitamin D serum levels reflect conditions associated with poor clinical outcomes (low dietary intakes, low solar exposure, obesity, etc.) but are not causative for cardiovascular diseases. On the other hand, whether high serum levels of vitamin D would increase vascular calcification in clinical settings is still debated. Of note, in NHANES III, a U-shaped relationship between vitamin D and mortality risk was described, with an increase in mortality when 25-OH vitamin D levels were >48 ng/L [25]. Carotid intima/media thickness and cardiac calcification scores had a bimodal distribution across vitamin D serum levels in a study comparing children on dialysis with controls [26].

There are few reports assessing the effects of supplemental calcium on cardiovascular outcomes. Some meta-analyses evidenced that supplemental calcium was associated with an increased risk of cardiovascular events [27]. A limitation of many studies is the fact that calcium and vitamin D are usually prescribed together and may exert a synergistic role.

One of the strengths of our model is the use of a relatively moderate dose of vitamin D and calcium, in a long-term follow-up. As expected, no vascular calcification was observed in wild-type animals but vitamin D and/or calcium supplementation accelerated the development of vascular calcification in animals affected by pyrophosphate deficiency, suggesting that even mild and intermittent increase in calcium phosphate supersaturation is sufficient to accelerate the calcification process in these models. It seems unlikely, in the view of the current literature, that vitamin D or calcium may have promoted specific local inflammatory processes but the link between calcification and inflammation in arteries deserves further specific studies in Abcc6^−/−^ mice. One limitation of our study is that the quantification of kidney calcification may in theory include some tubular plugs made of calcium phosphate, not exclusively vascular lesions. Actually, this may explain why, unlike in the aorta, increased calcium intake alone is sufficient to significantly aggravate kidney calcification. Nevertheless, histological examination of kidneys evidenced mainly vascular calcification in all cortical areas. PXE patients are probably not particularly prone to develop chronic disease but it remains unknown whether these patients may be affected by renal arterial calcification. This deserves further studies based upon high-resolution CT-scans of the kidneys in human cohorts.

These observations raise concerns about the prescription of vitamin D and/or calcium to patients affected by PXE. Interestingly, a report previously showed that vitamin D administration in PXE patients exacerbated skin calcifications, suggesting that vitamin D might increase soft tissue calcifications [28]. To a larger extent, patients with low pyrophosphate levels, including CKD patients, could be more at risk to develop vascular calcification when exposed to calcium and/or vitamin D supplementation.

## 4. Materials and Methods

### 4.1. Animal Studies

Mice knock-out for the *Abcc6* gene and named Abcc6^tm1Aabb^ were produced on a 129/Ola background and then backcrossed into a C57Bl/6J more than 10 times in Professor A. Bergen’s laboratory. These mice are designated *Abcc6^−/−^* in the current manuscript [15].

Forty-eight 10-week old female mice were housed and bred at INSERM UMR S 1155 Mouse Facility, with a twelve-hour dark/light cycle, as previously described [15]. Mice received standard chow, containing 1000 UI vitaminD3/Kg and 1.05 % calcium ad libitum. Six Abcc6^+/+^ (Wild type-WT) and 6 Abcc6^−/−^ mice had free access to water containing 80 mg/l of calcium (control group). Six WT and 6 Abcc6^−/−^ mice had free access to water containing 2g of calcium chloride (calcium group). Considering that a mouse’s daily drinking intake is about 5 mL/day (C57Bl/6J, 25g), calcium daily intake was 10 mg/mouse/day or 0.4 mg/g/day in “calcium” groups. Six WT and 6 Abcc6^−/−^ mice received vitamin D (ergocalciferol 2500 UI/mouse, Sterogyl 15H, DB Pharma, La Varenne Saint-Hilaire, France) every 2 weeks, during 6 months by s.c. injection, and had free access to water containing 80 mg/l of calcium (vitamin D group). Six WT and 6 Abcc6^−/−^ mice received similar vitamin D injections and had free access to water containing 2 g/L of calcium (calcium + vitamin D group). For this specific study, as WT mice do not develop significant calcification, we did not consider WT mice exposed to calcium or vitamin D alone but only WT mice exposed to the standard diet and to calcium + vitamin D. One WT mouse exposed to calcium + vitamin D died during the protocol (N = 5 mice in this group) for an unidentified reason and one aorta was inappropriately collected at the sacrifice in the WT group exposed to a standard diet (N = 5 aortas in this group).

All studies were performed in accordance with the European Union and National Institutes of Health guidelines (Comité d’Ethique en Experimentation Charles Darwin C2EA-05). The project was authorized by the health ministry and local ethics committee (Authorization number #11420 2017092015335292).

### 4.2. Biological Samples and Biochemistry

Urine was collected in metabolic cages as previously described, before the first administration of vitamin D and at 3 and 6 months [15]. Mice had free access to water (calcium-enriched or not). Mice were sacrificed 14 days after the last injection of vitamin D and blood was collected at that time.

The blood samples were analyzed for calcium (total), phosphate, urea, and vitamin D (at 6 months).

Calcium serum levels were measured with the Perkin-Elmer 3300 atomic absorption spectrometer. Vitamin D serum levels have been measured by the IDS-ISYS 25-Hydroxy Vitamin D immunoassay (IS-2700S), to assess 25-Hydroxy Vitamin D (D2 and D3) levels. Pyrophosphate urine levels were measured by using an ATP sulfurylase (M0394, NEB) to convert pyrophosphate into ATP in the presence of an excess of adenosine 5′phosphosulfate (A5508; Sigma Aldrich, Waltham, MA, USA). Generated ATP was then quantified using the ATP Determination kit (ATPlite 6016941; Perkin Elmer).

### 4.3. X-ray Microtomography and 3-Dimensional Modeling

The aorta and left kidney were dissected, fixed in formaldehyde, and embedded in paraffin. X-ray CT imaging was performed using a Skyscan 1272 (Bruker, Anvers, Belgium) at Lariboisière Hospital imaging platform (Paris, France). A 6 µm resolution scale was obtained. Shadow images were obtained using an X-ray energy of 65 kV and 150 mA without filter exposition. The angular step between image acquisitions was 0.5° and each image was averaged after 2 frames. Data were reconstructed using Nrecon software (Bruker, Anvers, Belgium) and then exported into a 16-bit Tag Image File Format stack of virtual slices. The Mimics Innovation suite 20.0 (Materialise, Leuven, Belgium) was used for the quantification of calcification volume, and 3-dimensional modeling of aorta and kidney calcifications. In the kidney, the papillary and inner medullary areas were excluded to consider and quantify only cortical (and outer medulla) calcification.

### 4.4. Histology and Yasue Staining

Four µm sections of aorta and kidney tissues were performed after X-ray microtomography and stained by Yasue procedure to reveal tissue calcifications.

A Zeiss SUPRA55-VP scanning electron microscope with an energy-dispersive X-ray (EDX) spectrometer was used for direct microstructure observation. Images were obtained without any conductive coating on the sample. This field emission gun microscope can operate at 0.5–30 kV accelerating voltage. High resolution observations were obtained around 1 KV using two secondary electron detectors: an in-lens and an Everhart-Thornley detector Tissue sections (4 mm) were investigated with a SUPRA^TM^ 55VP field emission-scanning electron microscope. Measurements were made at low voltage (1.4 KeV) and without the usual deposits of carbon at the surface of the sample. EDX experiments were performed. FE-SEM was performed at 12 keV for calcium and phosphate cartography.

### 4.5. mIRage™ Infrared Microscopy

Chemical characterization of the pathological calcification at the nanometer scale was performed by a technique using mid-infrared photons named OPTIR. OPTIR measurements (spectra and images) were done on a mIRage™ Infrared Microscope (Photothermal Spectroscopy Corp., Santa Barbara, CA, USA). Samples were placed on low-e microscope slides (MirrIR, Kevley Technologies, Tienta Sciences, Indianapolis). To generate data with a high signal to-noise ratio, 20–50 spectra were collected in reflection mode, at 2 cm^−1^ spectral data points spacing through a 40×, 0.78 NA, 8 mm working distance Schwarzschild objective. The pump IR source was a pulsed, tunable four-stage QCL device, scanning from 800 to 1900 cm^−1^, and we used a CW 532 nm visible variable power probe laser. A Density Functional Theory-Dispersion corrected (DFT-D) IR spectrum was calculated.

### 4.6. Statistical Analysis

Data are expressed as means +/− SEM. They were analyzed with non-parametric tests (Mann-Whitney) using Statview (SAS Institute, Cary, CA, USA) and GraphPad Prism software version 6 and 7 (GraphPad Software, San Diego, CA, USA). The level of significance was set at *p* < 0.05.

## 5. Conclusions

Our observations sound as a warning regarding the prescription of vitamin D, especially in synergy with high calcium intakes, in patients affected by PXE or other diseases characterized by pyrophosphate deficiency (GACI and ACDC). The dose of vitamin D used in our long-term experimental model was extremely moderate in comparison with preliminary murine models and did not significantly modify calcium serum and urine levels. There is a need to address in clinical settings whether vitamin D and/or calcium supplementation may accelerate the dramatic vascular calcifications affecting PXE (and GACI/ACDC) patients. To a larger extent, one may hypothesize that subjects with relatively low circulating pyrophosphate levels may be more at risk to develop arterial calcification, especially in response to a calcium-rich diet and/or vitamin D supplementation, deserving specific studies.
